# The value of AI-assisted multiple bone age assessment methods for evaluating efficacy in idiopathic short stature: A retrospective study of traditional Chinese medicine treatment for girls in China

**DOI:** 10.1097/MD.0000000000046413

**Published:** 2026-05-12

**Authors:** Wei Wang, Risheng Yu, Yue Hu, Di Le, Pingping Shen, Zhifeng Wang

**Affiliations:** a Department of Radiology, Ningbo Municipal Hospital of Traditional Chinese Medicine (TCM), Affiliated Hospital of Zhejiang Chinese Medical University, Ningbo, Zhejiang, China; b Department of Radiology, Second Affiliated Hospital, School of Medicine, Zhejiang University, Hangzhou, China.

**Keywords:** AI-assisted diagnosis, BA assessment, Greulich and Pyle atlas, idiopathic short stature, TW3, Zhonghua 05 method

## Abstract

There are few studies comparing the impact of different bone age (BA) assessment methods on treatment follow-up cycles. This study aimed to analyze the value of artificial intelligence-assisted multiple BA assessment methods in evaluating the efficacy of idiopathic short stature (ISS) in traditional Chinese medicine treatment. We selected 388 cases of healthy children’s BA data from November 2021 to August 2024 in a single center as the control group, and 190 cases of female pediatric patients with ISS admitted in the same period as the treatment group, which were divided into 120 cases in the effective group and 70 cases in the ineffective group according to the final therapeutic efficacy. They were divided into 3 periods of 6, 9, and 12 months according to the interval of follow-up. All the children underwent left hand X-ray examinations before treatment and after follow-up, all the X-rays were taken using artificial intelligence-assisted Greulich and Pyle atlas (G&P), Tanner Whitehouse 3 (TW3), and Tanner Whitehouse China 05 (TW C) methods for assessment. We analyzed the initial and follow-up BA assessment results and their differences and compared the relationship between the BA difference and the efficacy of each method. The results showed that the overall difference in BA follow-up was significantly higher in the treatment-effective group than in the ineffective group (*P* < .01). The assessment value of BA difference based on TW3 and TW C methods was better than that of the G&P method in the follow-up of the 6th, 9th and 12th months (*P* < .01). The TW C and TW3 methods could evaluate the efficacy of the treatment early in the 6-month follow-up, while the G&P method had a diagnostic value in the 12-month follow-up. Both TW3 and TW C methods of BA assessment can be used to evaluate the treatment effect as early as the 6-month follow-up period, and the TW C method had a higher diagnostic value of efficacy in Chinese girls with ISS compared with the TW3 and G&P methods.

## 1. Introduction

Bone age (BA), also known as skeletal age, refers to the method of measuring biological age by using the emergence, closure, and morphological changes of the ossification center of the body, which is widely used in the fields of adolescent growth and development, sports selection, and forensic science, etc. However, at present, there is no gold standard for the BA assessment,^[1]^ and the BA is affected by factors such as race, geography, economic level, and elevation, etc.^[2]^ There is a certain variability in the diagnostic results of BA among the different methods of assessment,^[3]^ and the accuracy of BA diagnosis is related to the diagnosis of adolescent growth and development diseases and the assessment of therapeutic effects.

Short stature is defined as an individual’s height being 2 standard deviations (2SD) below the mean or below the 3rd percentile of the average height of healthy children of the same age, sex, and race, which may be a phenotype of a clinical disease, and is generally categorized as pathologic short stature and normal variant short stature.^[4,5]^ The prevalence of children with short stature in China is 3.2%,^[6]^ and the most common causes are growth hormone deficiency and idiopathic short stature (ISS).^[7]^ ISS belongs to the general term of short stature of unknown etiology, and short stature seriously affects the physical and mental health of children and their adulthood.^[8]^ Currently, there is no standardized criteria for the treatment of ISS patients, Chinese expert consensus on the diagnosis and treatment of children with ISS (2023) recommends recombinant human growth hormone (rhGH) treatment for children with ISS diagnosis and height standard deviation score (Ht SDS) < -2.0,^[9]^ but the long-term effect of rhGH treatment on children’s growth is still uncertain, so most patients with ISS are still treated with basic therapy, a meta-study pointed out that traditional Chinese medicine (TCM) has a certain therapeutic efficacy in the treatment of ISS,^[10]^ due to the fact that the assessment and diagnosis of this kind of disease are often late and the treatment cycle of ISS is long, and children’s growth spurt period is short, long-term ineffective treatment may miss the optimal intervention period for children’s growth disorder diseases, early and accurate evaluation of the efficacy of TCM in treating ISS is crucial and essential, some ISS patients’ BA lags behind children of the same age by more than 1 year, and BA is highly correlated with the SD score for child height (Ht SDS) and predicted adult height.

Therefore, in this study, we aimed to compare the BA and follow-up period difference between healthy children and girls with ISS using the Greulich and Pyle atlas (G&P), Tanner Whitehouse 3 (TW3), and Tanner Whitehouse China 05 (TW C) methods, to explore the value of TCM combined with basic treatment for ISS patients in the assessment of efficacy at each follow-up period.

## 2. Materials and methods

### 2.1. General material

**Figure 1.**
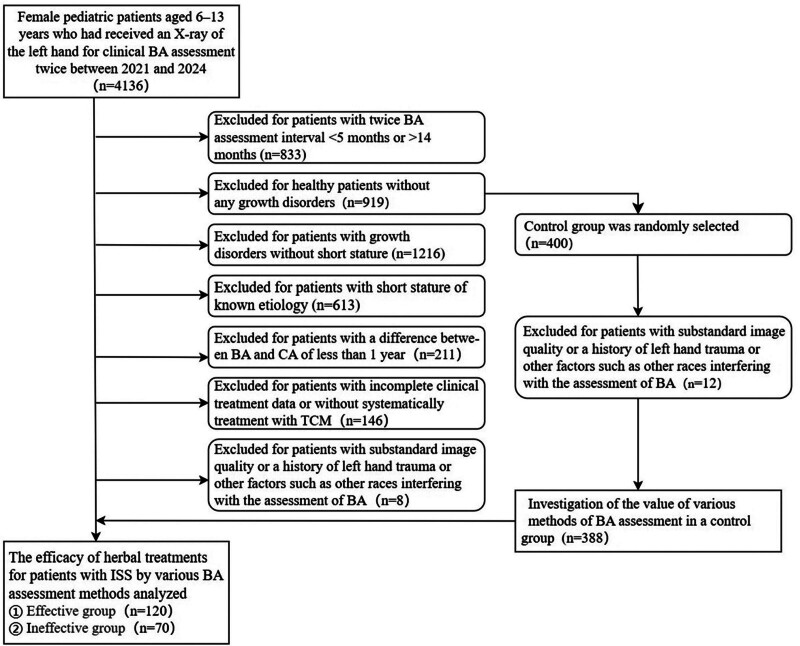
Flowchart for selection of treatment and control groups. BA = bone age, CA = chronological age, TCM = traditional Chinese medicine, ISS = idiopathic short stature.

The main sign to suspect the onset of puberty is breast tissue development (thelarche) in girls,^[11]^ and the difference in annual growth rate between prepubertal and pubertal children has a great impact on the assessment of the efficacy of ISS patients, and it is difficult to determine the time when boys enter puberty. Therefore, in this study, 190 BA X-rays before and after treatment of girls with ISS who were treated at Ningbo Municipal Hospital of TCM from October 2021 to August 2024 were selected as study subjects (Fig. 1). Based on the final efficacy, the patients were divided into treatment-effective group (n = 120) and treatment ineffective group (n = 70). All children with short stature were diagnosed with ISS after excluding all known etiologies during the initial and follow-up consultations, and received BA X-rays during the initial and follow-up consultations, and randomly selected BA X-rays of 388 healthy children who visited our hospital during the same period of time, and all of them contained follow-up X-rays of BA within 1 year.

This was a retrospective study, which followed the principles of the Declaration of Helsinki and was approved by the Ethics Committee of Ningbo Municipal Hospital of TCM (approval number: 2025-016-01). In this study, we dispensed the requirement for obtaining written informed consent from the patients by de-identifying the imaging data.

### 2.2. Criteria of inclusion and exclusion

Inclusion criteria: all children met the diagnosis of ISS, and the diagnostic criteria were based on the Chinese Expert Consensus on the Diagnosis and Treatment of Idiopathic Short Stature in Children (2023).^[9]^ All children were treated for the 1st time, and did not use rhGH analogs prior to the treatment. The age of the children ranged from 6 to 13 years. Height and weight were in the normal range at the time of birth. The control group comprised children who attended the clinic for health checkups.

Exclusion criteria: serious congenital diseases and/or liver or kidney dysfunction. Suffering from chromosomal diseases, inherited metabolic diseases or thyroid dysfunction and growth retardation caused by all kinds of chronic diseases. History of obvious trauma or wrist deformity. Combined bone diseases and rickets. Age less than 6 years or more than 13 years. Bone age delay not more than 1 year.

### 2.3. Treatment and criteria for the assessment of efficacy

All patients enrolled in the treatment group received 6 to 8 weeks of TCM treatment within 3 months of consultation. The effective diagnosis criteria of the treatment were as follows: height growth < 4 cm/year (pre-puberty) or < 6 cm/year (puberty) at the initial consultation, and BA lagged behind the average value of children of the same gender and age for more than 1 year, and after systematic TCM treatment, the annual growth of height was > 4 cm/year (pre-puberty) or > 6 cm/year (puberty) at the follow-up consultation.

### 2.4. Examination methods and image data processing

Left hand posterior–anterior radiographs were obtained using a Dutch Philips C50 digital X-ray machine (DR) with tube voltage of 40 to 60 kV, tube current of 2 to 5 mAs, and a focal length of 90 cm, the scope of radiographs included the carpal bones, metacarpophalangeal bones, and distal segment of the radial-ulnar trunk of 3 to 4 cm. All the image data were uploaded to the PACS system, and the image data were in the format of Dicom.

### 2.5. Bone age assessment methods

Two attending physicians of Ningbo Municipal Hospital of TCM used Deepwise Medical Bone Age AI diagnostic software (version: 1.23.08.31, China) to assess BA. The G&P method of BA referred to the Bone Age Atlas published by Stanford University Press,^[12]^ and the TW C scoring method of BA referred to the “Chinese Children’s Bone Age Scoring Method”^[13]^ and the “Chinese People’s Wrist Bone Age Criteria.”^[14]^ After reviewing and proofreading the artificial intelligence (AI) diagnostic results, the average value was taken as the final result of BA assessment, and a total of 5 categories of BA results diagnosed by the 3 methods of G&P, TW3 (TW3-RUS, TW3-Carpal), and TW C (TW C-RUS and TW C-Carpal) were obtained. Among them, the applicable range of TW3-Carpal BA for girls is from 1 year and 7 months to 13 years old, and the applicable range of TW C-Carpal BA for girls is from 3 years to 11 years and 6 months old, and both TW3-Carpal and TW C-Carpal BA beyond this range are considered as developmental maturity and treated as defective value.

The TW C method has been improved by Chinese scholars based on the TW3 method; the assessment of metacarpophalangeal and carpal bones is the same as that of the TW3 method. According to the developmental characteristics of children in China, the classification of bone development grades is more detailed, and the weighted calculation of the scores of some bone maturity is different from that of TW3.

### 2.6. Observational index

The treatment group underwent X-ray imaging of the left hand of the girl before and after treatment, while the control group underwent X-ray imaging during two consecutive visits, including the carpal bones, metacarpophalangeal bones, and distal segment of the radial-ulnar trunk, and recorded the BA, and then calculated the BA difference (BAD) and the follow-up period (F/U P) of the various BA assessment modalities, respectively. Because the follow-up was not strictly according to the specified time, we defined the control group and treatment group according to the follow-up interval of 5 to 7 months as the 6-month follow-up period group, 8 to 10 months as the 9-month follow-up period group, and 11 to 13 months as the 12-month follow-up period group.

### 2.7. Statistical methods

SPSSAU (Version 25.0; Online Application Software, Beijing, China: Qingsi Technology) was used in this study.^[15]^ The ICC was used to evaluate the consistency of the quantitative BA assessment data between the 2 physicians. Descriptive statistics and comparison of means were applied to the BA data, and *t* tests were performed. Normality was tested using the Kolmogorov–Smirnov test for quantitative data, and conformity to normal distribution measurements were expressed as mean ± standard deviation ($\bar{x}\pm s$). Five categories of 3 BA methods were compared using 1-way ANOVA, Spearman correlation was used to analyze BA and chronological age (CA) correlation, scatter plots were drawn. Various BA assessment methods for treatment efficacy were tested using logistic regression, ROC curves were used to evaluate the diagnostic value of different BA assessment methods for efficacy, and independent samples were tested for differences using the Hanley & McNeil method. Differences in the efficacy of treatment groups were analyzed using the Mann–Whitney test, statistic and differences in diagnostic efficacy were compared using the *Z* test. *P* < .05 was considered statistically significant.

## 3. Results

### 3.1. Consistency analysis

Two attending physicians independently assessed 60 radiographs using 5 BA assessment methods, and the results of BA assessed by the 2 physicians were not statistically significant. (*P* > .05).

### 3.2. Comparison of initial visits of BA assessments and CA in the control group

The mean age at initial visits of the children in the control group (9.816 ± 1.247) years with a median of 9.96 years, and the mean values of the BA diagnosis of G&P, TW3-RUS, TW3-Carpal, TW C-RUS, and TW C-Carpal ranged from 9.415 to 9.884 years, with a median between 9.58 and 10 years, and a 95% confidence interval (CI) of the mean values between 9.294 and 10.041 years (Table 1).

**Table 1:** Results of initial visits of chorological age (CA) and bone age (BA) assessment in the control group ($\bar{x}\pm \;s$, year).

Category	Cases (n)	Min	Max	Mean ± SD	VAR	M	SE	Mean 95% CI (LL)	Mean 95% CI (UL)
CA	388	6.5	13.08	9.816 ± 1.247	1.556	9.96	0.063	9.692	9.94
G&P	388	5.67	14.67	9.792 ± 1.654	2.737	9.83	0.084	9.627	9.957
TW3-RUS	388	6.08	14	9.884 ± 1.578	2.491	9.915	0.08	9.727	10.041
TW3-Carpal	374	6.17	12.17	9.826 ± 1.169	1.366	10	0.06	9.708	9.945
TW C-RUS	388	6.25	12.83	9.857 ± 1.252	1.567	10	0.064	9.732	9.981
TW C-Carpal	366	6	11.33	9.415 ± 1.183	1.401	9.58	0.062	9.294	9.536

G&P = Greulich and Pyle atlas, TW C = Tanner Whitehouse China 05, TW3 = Tanner Whitehouse 3.

### 3.3. Relationship between the follow-up period and the difference between the results of BA assessments in the control group

**Figure 2.**
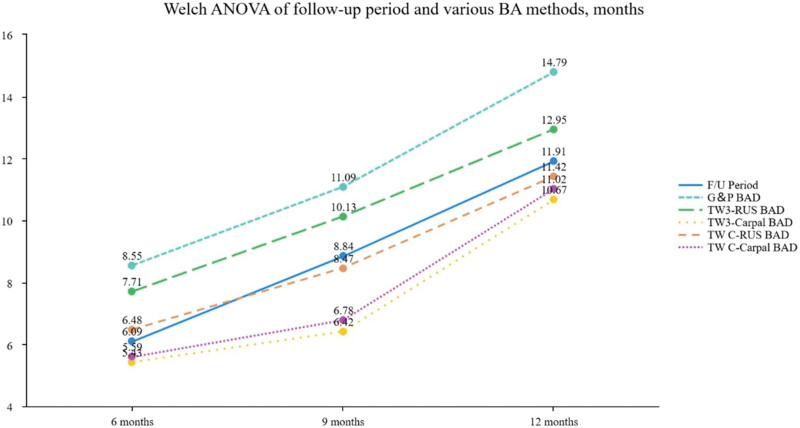
Welch ANOVA of follow-up period and various bone age methods, months. BAD = bone age difference, F/U period = follow-up period.

The difference between the results of different follow-up periods and the results of each BA assessment method in the control group were significantly different (*P* = .000) (Table 2, Fig. 2). By comparing *F*-value, mean and SD, TW C-RUS had the best diagnostic results in 6-, 9- and 12-months follow-up periods with the BA assessment results of (6.48 ± 1.86) months, (8.47 ± 1.96) months, and (11.42 ± 2.34) months, respectively. Next was TW3-RUS with the BA assessment results of (7.71 ± 2.60) months, (10.13 ± 2.45) months, and (12.95 ± 3.02) months, respectively. Again G&P method, the BA assessment results were (8.55 ± 4.05) months, (11.09 ± 4.47) months, and (14.79 ± 4.22) months. The worst were TW3-Carpal and TW C-Carpal methods. Therefore, G&P, TW3-RUS, and TW C-RUS methods were chosen in this study to further investigate the efficacy of different BA assessment methods in the treatment group.

**Table 2:** Normality test of chorological age and bone age results in the control group.

Category	Cases (n)	Mean	SD	Kurtosis	Skewness	Kolmogorov–Smirnov test	
						Statistic *D*-value	*P*
CA	388	9.816	1.247	-0.311	-0.040	0.080	.000**
G&P	388	9.792	1.654	-0.069	-0.390	0.060	.002**
TW3-RUS	388	9.884	1.578	-0.158	-0.245	0.049	.025*
TW3-Carpal	374	9.826	1.169	-0.612	0.108	0.091	.000**
TW C-RUS	388	9.857	1.252	-0.440	0.023	0.083	.000**
TW C-Carpal	366	9.415	1.183	-0.579	-0.321	0.120	.000**

BA = bone age, CA = chronological age, G&P = Greulich and Pyle atlas, TW C = Tanner Whitehouse China 05, TW3 = Tanner Whitehouse 3.

* *P* < .05.

** *P* < .01.

### 3.4. Comparison of initial visits of BA assessments and CA in the treatment group

CA of the treatment group at the initial visits were (9.981 ± 1.652) years old with a median of 9.875 years old, and BA of the treatment group at the initial visits by G&P, TW3-RUS, and TW C-RUS methods were (8.536 ± 2.082) years old, (8.572 ± 2.013) years old, and (8.710 ± 1.732) years old, with a median of 7.92 years old, 8.17 years old, and 8.5 years old, respectively, based on each BA assessment, the mean and median BA in the treatment group were more than 1 year behind the CA (Table 3).

**Table 3:** Results of initial visits of CA and BA assessment in the treatment group ($\bar{x}\pm \;s$, years).

Category	Cases (n)	Min	Max	Mean ± SD	VAR	M	SE	Mean 95% CI (LL)	Mean 95% CI (UL)
CA	190	6.5	13.33	9.981 ± 1.652	2.729	9.875	0.12	9.746	10.216
G&P	190	4.5	13.58	8.536 ± 2.082	4.335	7.92	0.151	8.24	8.832
TW3-RUS	190	4.67	13.08	8.572 ± 2.013	4.053	8.17	0.146	8.286	8.859
TW C-RUS	190	5.17	12.08	8.710 ± 1.732	3.000	8.5	0.126	8.464	8.957

BA = bone age, CA = chronological age, G&P = Greulich and Pyle atlas, TW C = Tanner Whitehouse China 05, TW3 = Tanner Whitehouse 3.

### 3.5. Relationship between follow-up period, difference of each BA assessment and efficacy in the treatment group

**Figure 3.**
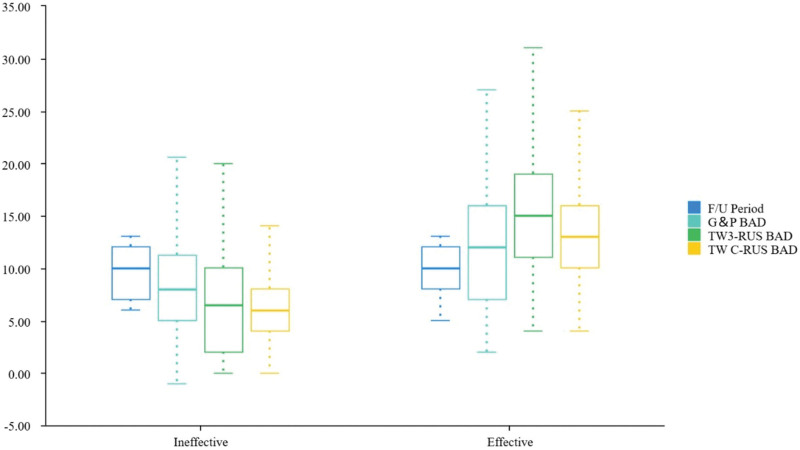
Box line plot of follow-up period and bone age difference versus efficacy in the treatment group, months. BAD = bone age difference, F/U period = follow-up period.

We further investigated the relationship between the difference of each BA assessment method and the variability of efficacy in the follow-up period of the treatment group by performing a non-parametric test on the samples (Table 4) and constructing a box line plot of the test results (Fig. 3), and using the Mann–Whitney statistic on the data of ineffective versus effective groups in the treatment group, which showed that the difference of diagnosis of BA by G&P, TW3-RUS, and TW C-RUS method had a statistically different (Z = -3.873, -8.537, -9.115, *P* < .001, *P* < .001, *P* < .001), and when comparing the median difference between the G&P, TW3-RUS, and TW C-RUS ineffective and effective groups, the ineffective group was significantly lower than the effective group.

**Table 4:** Non-parametric test analysis of the difference between BA difference and effective in treatment groups.

	Effective (M, mo)		Mann–Whitney test *U* value	Mann–Whitney test *Z*-value	*P*
	No (n = 70)	Yes (n = 120)			
F/U P	10.000	10.000	4129.500	-0.195	.846
G&P	8.000	12.000	2787.000	-3.873	.000*
TW3-RUS	6.500	15.000	1082.000	-8.537	.000*
TW C-RUS	6.000	13.000	875.000	-9.115	.000*

BA = bone age, G&P = Greulich and Pyle atlas, TW C = Tanner Whitehouse China 05, TW3 = Tanner Whitehouse 3.

* *P* < .01.

### 3.6. The value of the diagnostic efficacy of each BA assessment method in the treatment group

**Figure 4.**
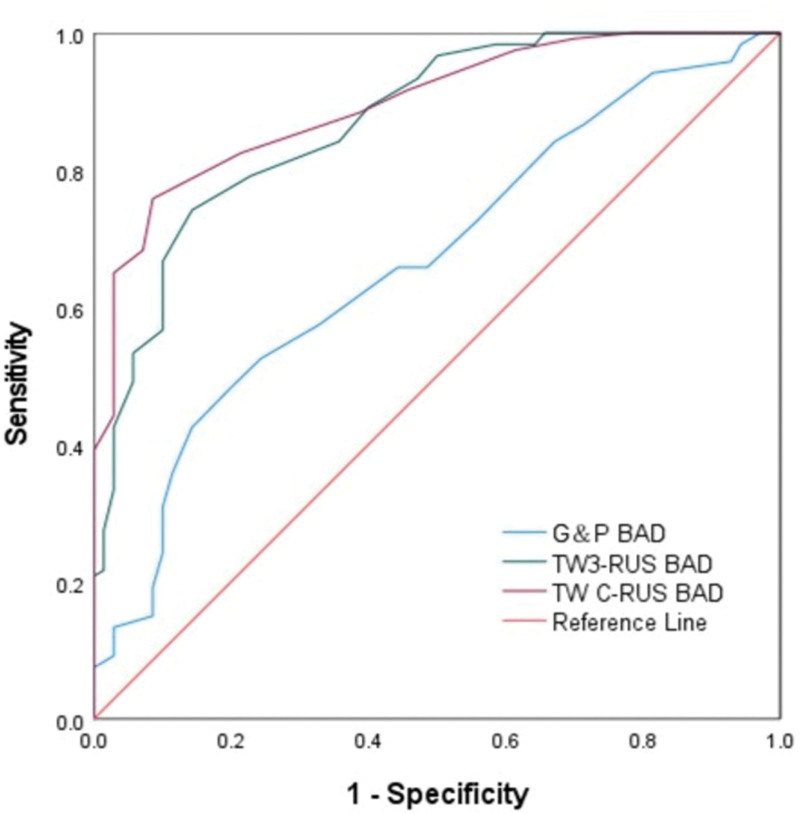
ROC curves for the overall efficacy diagnostic value of G&P, TW3-RUS, and TW C-RUS methods in the treatment group. The areas under the curves were 0.668, 0.871, and 0.896. BAD = bone age difference, G&P = Greulich and Pyle atlas, TW C = Tanner Whitehouse China 05, and TW3 = Tanner Whitehouse 3.

ROC curves were constructed based on the difference between the BA assessment methods and the final efficacy for all patients enrolled in the treatment group (Fig. 4), and AUC values were used to compare the value of each BA assessment method for efficacy diagnosis (Table 5).

**Table 5:** Summary of AUC for total validity ROC results for each bone age assessment method in the treatment group.

Method	AUC	SE	*P*	95% CI
G&P	0.668	0.040	.000*	0.590–0.746
TW3-RUS	0.871	0.026	.000*	0.820–0.922
TW C-RUS	0.896	0.022	.000*	0.853–0.939

G&P = Greulich and Pyle atlas, TW C = Tanner Whitehouse China 05, TW3 = Tanner Whitehouse 3.

* *P* < .01.

The AUC values corresponding to the G&P method BAD were 0.668 (95% CI: 0.590–0.746), the AUC values corresponding to the TW3-RUS method BAD were 0.871 (95% CI: 0.820–0.922), and the AUC values corresponding to the TW C-RUS method BAD were 0.896 (95% CI: 0.853–0.939).

Delong test of ROC curves using the Hanley & McNeil method for G&P, TW3-RUS, and TW C-RUS of BAD in the treatment group (Table 6) showed that there was a difference in the G&P versus TW3-RUS and TW C-RUS diagnostic methods (*P* < .01), and there was no difference in the TW3-RUS and TW C-RUS diagnostic methods (*P* > .05).

**Table 6:** Delong test of ROC curves for overall G&P, TW3-RUS, and TW C-RUS of bone age differences in the treatment group.

First difference	Second difference	AUC difference	SE	95% CI	*Z*-value	*P*-value
G&P	TW3-RUS	0.2030	0.0361	0.132 to 0.274	5.6224	.0000
G&P	TW C-RUS	0.2276	0.0362	0.157 to 0.299	6.2847	.0000
TW3-RUS	TW C-RUS	0.0246	0.0233	-0.021 to 0.070	1.0564	.2908

G&P = Greulich and Pyle atlas, TW C = Tanner Whitehouse China 05, TW3 = Tanner Whitehouse 3.

**Figure 5.**
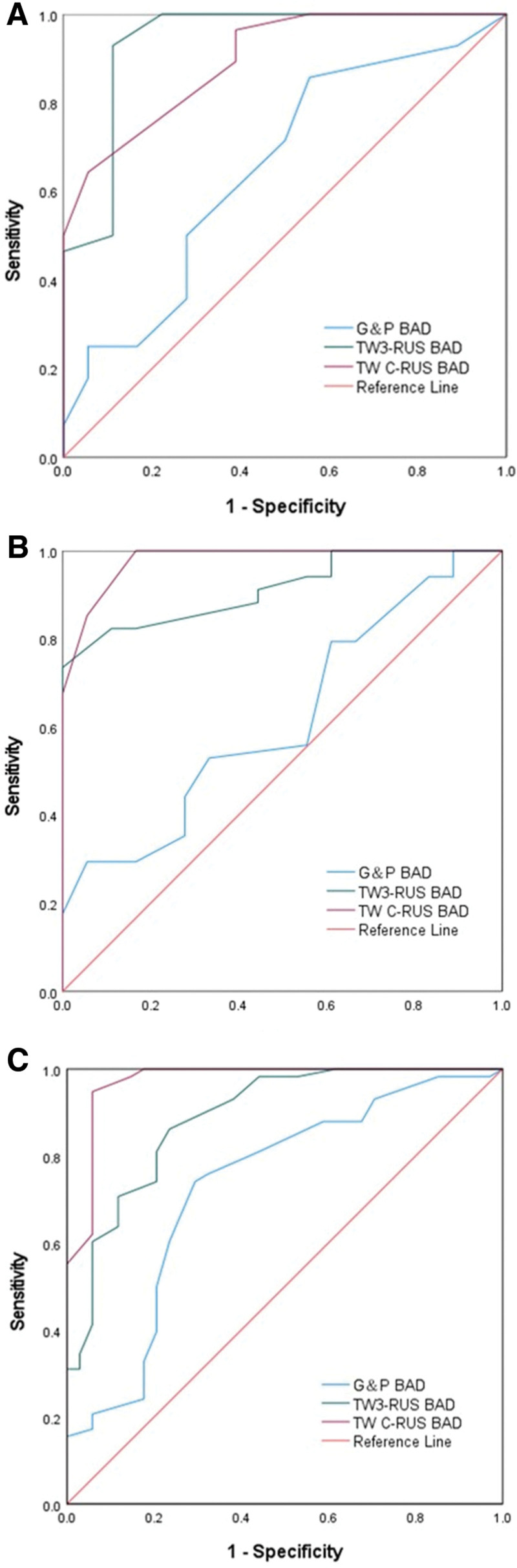
A ROC curves for the diagnostic value of efficacy at 6 months for G&P, TW3-RUS, and TW C-RUS methods. The areas under the curves were 0.652, 0.938, and 0.896. (B) ROC curves for the diagnostic value of efficacy at 9 months for G&P, TW3-RUS and TW C-RUS methods. The areas under the curves were 0.619, 0.913, and 0.979. (C) ROC curves for the diagnostic value of efficacy at 12 months for G&P, TW3-RUS, and TW C-RUS methods. The areas under the curves were 0.731, 0.890, and 0.972. BAD = bone age difference, G&P = Greulich and Pyle atlas, TW C = Tanner Whitehouse China 05, TW3 = Tanner Whitehouse 3.

Further, we investigated the value of each BA assessment method for the diagnosis of efficacy in each follow-up period (Fig. 5A–C, Tables 7–9).

**Table 7:** AUC results of the ROC curves for the diagnostic value of each bone age difference in the treatment group at 6 months follow-up.

Method	AUC	SE	*P*	95% CI
G&P	0.652	0.084	.085	0.487–0.817
TW3-RUS	0.938	0.041	.000*	0.859–1.000
TW C-RUS	0.896	0.045	.000*	0.809–0.983

G&P = Greulich and Pyle atlas, TW C = Tanner Whitehouse China 05, TW3 = Tanner Whitehouse 3.

* *P* < .01.

**Table 8:** AUC results of the ROC curves for the diagnostic value of each bone age difference in the treatment group at 9 months follow-up.

Method	AUC	SE	*P*	95% CI
G&P	0.619	0.080	.160	0.462–0.777
TW3-RUS	0.913	0.038	.000*	0.839–0.987
TW C-RUS	0.979	0.017	.000*	0.946–1.000

G&P = Greulich and Pyle atlas, TW C = Tanner Whitehouse China 05, TW3 = Tanner Whitehouse 3.

* *P* < .01.

**Table 9:** AUC results of the ROC curves for the diagnostic value of each bone age difference in the treatment group at 12 months follow-up.

Method	AUC	SE	*P*	95% CI
G&P	0.731	0.057	.000*	0.620–0.842
TW3-RUS	0.890	0.035	.000*	0.821–0.959
TW C-RUS	0.972	0.018	.000*	0.938–1.000

G&P = Greulich and Pyle atlas, TW C = Tanner Whitehouse China 05, TW3 = Tanner Whitehouse 3.

* *P* < .01.

The ROC curves were constructed for the G&P BAD, TW3-RUS BAD, and TW C-RUS BAD at the 6-, 9-, and 12-month follow-up periods, respectively, in which the AUC values of the G&P method at the 6-, 9-, and 12-month follow-up periods were 0.652 (*P* = .085), 0.619 (*P* = .160), 0.731 (*P* < .001); AUC values for the TW3-R method were 0.938 (*P* < .001), 0.913 (*P* < .001), 0.890 (*P* < .001) in the 6-, 9-, and 12-month follow-up periods, respectively; AUC values for the TW3-R method were 0.938(*P* < .001), 0.913 (*P* < .001), and 0.890 (*P* < .001). The TW3-R and TW C-R methods were statistically significant for the assessment of efficacy in the 6-, 9-, and 12-month follow-up periods (*P* < .001), and the G&P method was not statistically significant for the diagnosis of efficacy at the 6- and 9-month follow-up periods (*P* > .05), and statistically significant for efficacy diagnosis in the 12-month follow-up period (*P* < .001).

## 4. Discussion

BA assessment is frequently utilized in child growth and development clinics for the evaluation of children’s growth and development, as well as to assist in the diagnosis of specific growth and development disorders.^[16]^ Additionally, BA assessment is often employed as an indicator of treatment efficacy for associated diseases.^[17]^ However, there is a paucity of studies that directly compare the values of different BA assessment methods in the treatment of the same disease. In this study, we compared several commonly used BA assessment methods, and finally selected the G&P, TW3-RUS, and TW C-RUS BA assessment methods to evaluate the treatment effect of ISS patients, and investigated the value of each BA diagnostic method in the evaluation of the therapeutic effect of ISS in female pediatric patients. The findings of this study indicate that TW3-RUS and TW C-RUS BA are suitable for the evaluation of short- and medium-term efficacy. These assessments have the potential to provide valuable clinical insights into the diagnosis of efficacy at 6 and 9 months of treatment.

In the control group, the differences in the G&P, TW3 and TW C results were compared during the follow-up period. TW C-RUS had a standard deviation of 2 months or less in the 6- and 9-month follow-up period, and in the 12-month follow-up period, the standard deviation was 2.34 months, and the mean values of each follow-up subgroup were close to the follow-up period and less than 1 month or less, followed by TW3-RUS in the 6-month, 9-month follow-up period, the standard deviation was within 2.6 months, and in the 12-month follow-up, the standard deviation was in 3.02 months, but the mean values were all greater than 1 month or more than the follow-up period, while the G&P, TW3-Carpal, and TW C-Carpal mean and standard deviation in each follow-up subgroup with the follow-up period difference were larger, the G&P method short-term difference of the larger results we consider as a reference atlas to compare the BA, the diagnosis of BA results is a general range, may be small metacarpal phalangeal developmental variations, the physician is more inclined to diagnose it as a higher BA results, while analyzing the results of TW3-Carpal and TW C-Carpal methods, the mean values were close to the follow-ups while the standard deviations were large, which may be related to the greater variability of carpal bone development.^[18]^

In the treatment group, the AUC value of the G&P method was below 0.7 in the 6-month and 9-month follow-up periods, which had no diagnostic value of efficacy in that follow-up period, and its AUC value was 0.731 (95% CI: 0.620–0.842) during the 12-month follow-up period, which had a higher diagnostic value of efficacy. The TW3-RUS and the TW C-RUS methods had diagnostic value of efficacy in all the follow-up periods. Among them, the AUC value of TW3-RUS method was greater than 0.9 in 6- and 9-month follow-up period, but the AUC value was 0.890 (95% CI: 0.821–0.959) in 12-month follow-up period. The TW C-RUS method had AUC values of 0.979 (95% CI: 0.946–1.000) and 0.972 (95% CI: 0.938–1.000) at the 9- and 12-month follow-up periods, respectively. Specifically, the AUC value of the TW3-RUS method gradually decreased with the prolongation of the follow-up period, while the AUC value of the TW C-RUS method gradually increased with the prolongation of the follow-up period and was maintained at the level of 0.97, which may be related to the shorter follow-up period of 6 and 9 months in the treatment group and the error in the assessment of BA, and may also be related to the statistical bias arising from the small number of cases. For the overall analysis of all cases in the treatment group, the AUC value corresponding to the difference in BA of TW3-RUS was 0.871 (95% CI: 0.820–0.922) and the AUC value corresponding to the difference in BA of TW C-RUS was 0.896 (95% CI: 0.853–0.939) in the treatment group, which showed a better performance in the efficacy evaluation of the TW C-RUS method compared to the TW3-RUS method.

BA assessment methods include atlas and scoring methods. Although the internationally dominant G&P atlas and TW2/3 methods are widely used worldwide,^[19]^ the applicability of their BA databases established based on Caucasian populations in the last century to populations other than Europe and America has been questioned.^[20,21]^ Our scholars established the modified TW3 method–TW China method (TW C, Zhonghua 05 method) based on BA data of urban children in eastern China, which demonstrated superior local adaptability.^[22]^ However, although the G&P method has the advantages of simplicity and convenience, its assessment results are highly dependent on physicians’ experience, and the problem of insufficient reproducibility limits its application value in short-term treatment efficacy,^[23]^ and this study showed that the G&P method did not have a statistically significant effect on efficacy diagnosis in the 6- and 9-month follow-up periods. The TW2/3 scoring method quantitatively analyzes the maturity of specific bone development, and has the advantages of relative objectivity, reproducibility, and accuracy of BA assessment. However, it is time-consuming and requires the application of computer-assisted software, which is limited to outpatient application and is mostly used for research. The TW3 and TW C methods used in this study were assisted by the AI-BA assessment software, and the results were further checked by radiologists, which significantly shortened the diagnostic time of the scoring method, effectively balanced the needs of efficiency and accuracy, made the application of the scoring method more promising, and reduced the shortcomings of poor generalization of AI diagnostic results alone.

In 2009, Thodberg’s team proposed the BoneXpert^[24]^ BA assessment system, which became commercially available in Europe, and which was validated over the age range of 2 to 17 years on the G&P atlas, with a SD of 0.42 years (95% CI: 0.37–0.47). Automated AI algorithms have been suggested to improve the efficiency of clinical routines by reducing reading time without compromising accuracy compared to the G&P method.^[25]^ In a recent German study comparing the errors of three European commercial BA assessment software with expert assessments by means of the G&P-based method, the root-mean-square error ranged from 0.62 to 0.75 years and the standard deviation of the AI assessment results was lower than that of the expert assessment results.^[26]^ A multicenter prospective study further validated that the TW3-method AI-BA assessment system based on convolutional neural network improved the diagnostic efficiency and consistency characteristics of diagnostic results.^[27]^ Although the G&P and TW3 methods are still applicable to the assessment of BA in children in China, there is a significant bias in some age groups. With the popularization of computer-assisted AI software, the scoring method can be processed by the BA AI software to diagnose the results more quickly,^[28]^ and with a higher accuracy.^[29]^

There is an upper limit to the assessment of carpal bones in scoring methods such as TW3 and TW C,^[30]^ and there is variability in the development of BA between men and women; at the same time, BA is closely related to a variety of factors, and BA assessment methods with better consistency should be selected according to different races and ages. However, even with the scoring method, BA assessment is still a summary of the results of human visual judgments, so differences in BA assessment are inevitable. With the iterative development of BA AI algorithms, more extensive BA studies, and the establishment of larger BA databases, the accuracy of BA assessments will increase.

ISS belongs to children’s growth shortness disease of unknown etiology, there is no exact treatment, in addition to the basic treatment, some medical institutions are trying to use growth hormone,^[31–33]^ genetic testing,^[34]^ Chinese herbs^[10]^ and other diagnostic and therapeutic modalities to explore the treatment of idiopathic short stature, how to evaluate the efficacy of the various therapeutic modalities has always been plagued by clinical,^[35]^ the ultimate goal of treatment for patients with ISS is to improve lifelong height, and the development of BA is closely related to growth potential. Currently, the indicator of whether the treatment is effective is often evaluated using the change in height velocity and Ht SDS,^[36]^ but the short-term height velocity is affected by a number of factors^[37]^ such as the method of measurement, the time of measurement, the short-term fluctuation of the child’s own hormone levels, and exercise, therefore, our study proposes that BA assessment change profile can be a useful complementary indicator for short-term efficacy assessment of clinical treatments.

This study also has the following limitations: this study is a single-center study, the sample size used for efficacy assessment is small, and selection bias is difficult to avoid; this study only discusses ISS patients in girls, so the applicability to idiopathic short stature patients in boys needs to be further researched; as a retrospective study, based on previous treatment data and did not intervene homogeneously in the treatment process; therefore, prospective multicenter studies are needed to further validate the reliability of the study; whether or not BA is delayed may be related to the subtype of ISS, and this study lacks research on such patients without BA delay.

## 5. Conclusion

In conclusion, G&P, TW3, and TW C methods all have high diagnostic value for BA assessment in daily clinical application, TW3-RUS and TW C-RUS methods are recommended for short-term efficacy assessment of TCM treatment for girls with ISS, such as 6-, 9- and 12-month follow-ups, and G&P can be used for efficacy assessment during the 12-month follow-up period, and for Chinese female pediatric ISS patients, the TW C-RUS method is recommended, and the same method should be selected for BA assessment during follow-up.

## Author contributions

**Conceptualization**: Wei Wang, Risheng Yu, Yue Hu.

**Data curation**: Wei Wang, Yue Hu, Di Le.

**Formal analysis**: Wei Wang, Pingping Shen.

**Methodology**: Risheng Yu, Zhifeng Wang.

**Project administration**: Wei Wang, Zhifeng Wang.

**Resources**: Zhifeng Wang.

**Writing – original draft**: Wei Wang.

**Writing – review & editing**: Wei Wang, Risheng Yu, Zhifeng Wang.
